# Clinical characteristics, radiologic features, and histopathology of biopsied lacrimal gland tumors

**DOI:** 10.1038/s41598-023-43817-0

**Published:** 2023-10-03

**Authors:** Orapan Aryasit, Pawarin Amornthatree, Wantanee Dangboon Tsutsumi, Wantanee Sittivarakul, Alan Frederick Geater, Supaporn Tengtrisorn, Virintorn Prapakornkovit

**Affiliations:** 1 Department of Ophthalmology, Faculty of Medicine, Prince of Songkla University, 15 Kanjanavanich Road, Kohong, Hat Yai, 90110 Songkhla Thailand; 2 Epidemiology Unit, Faculty of Medicine, Prince of Songkla University, Kohong, Hat Yai, 90110 Songkhla Thailand

**Keywords:** Diseases, Health care, Medical research, Signs and symptoms

## Abstract

Herein, we described the clinicopathologic and radiologic features of biopsied lacrimal gland tumors. A retrospective case series of 79 patients treated between 2004 and 2021 was reviewed. The median age was 48.9 years (range 18.3–88.3 years), with 51.9% females. The histopathologic diagnoses were as follows: immunoglobulin G4-related disease (IgG4-RD) = 23, reactive lymphoid hyperplasia = 14, lymphoma = 14, nonspecific inflammation = 10, adenoid cystic carcinoma (ACC) = 9, and pleomorphic adenoma = 9. The proportion of histopathologic diagnoses did not differ significantly over the range of symptom durations (≤ 1 month, > 1–3 months, > 3 months). Patients with ACC had significantly shorter symptom duration and more frequent proptosis than those with pleomorphic adenoma (*p* = 0.040 and *p* = 0.009, respectively). Patients with IgG4-RD were older (median 54.3 years) than those with nonspecific inflammation (36.2 years; *p* = 0.046). Patients with ACC were more likely to present with diplopia than those with lymphoma (*p* < 0.001). The superior wedge sign increased the likelihood of ACC compared with that of non-epithelial non-malignant lacrimal gland tumors (relative risk ratio = 13.44, *p* = 0.002). The overall survival of patients with ACC and lymphoma did not differ significantly. Although these patients present with a short symptom duration, urgent orbital imaging, tissue biopsy, and prompt treatment should be performed in patients with lacrimal gland tumors.

## Introduction

Lacrimal gland tumors represent 22–35% of orbital space-occupying lesions^1–4^. The incidence of biopsied lacrimal gland lesions is 1.3 per million people per year^5^. According to a previous oft-quoted epidemiologic study from 1956, approximately 50% of lacrimal gland tumors are epithelial in origin, and the other half are non-epithelial tumors^6^. Although the majority of lesions are benign, a small proportion of aggressive malignancies are potentially sight- and life-threatening. Clinical and radiologic features are significant clues for diagnosis; however, some findings are vague, making the role of tissue biopsy in histopathologic diagnosis crucial for proper management. Most studies have reported the distribution of histopathologic diagnoses of lacrimal gland lesions in Western countries owing to the rarity of lacrimal gland lesions^7,8^. Few studies exist on the clinicopathologic and radiologic features of lacrimal gland tumors in Asian populations^9–11^.

Our center is a major referral tertiary hospital in southern Thailand with a large number of diverse cases. This study aimed to determine the clinicopathologic features, radiologic findings, and treatment outcomes of biopsied lacrimal gland tumors to help guide clinicians in diagnosis, provide proper management, and discuss prognosis.

## Methods

This retrospective study was approved by the Human Research Ethics Committee of the Faculty of Medicine, Prince of Songkla University (REC number 64-026-2-4). This study adhered to the principles of the Declaration of Helsinki. The requirement for informed consent was waived due to the retrospective nature of the study. The electronic medical records were reviewed retrospectively. Patients with lacrimal gland-occupying lesions who underwent incisional biopsy or excision between January 2004 and December 2021 were enrolled. We excluded patients with a follow-up period of less than 3 months and those for whom orbital imaging was not available.

**Figure 1:**
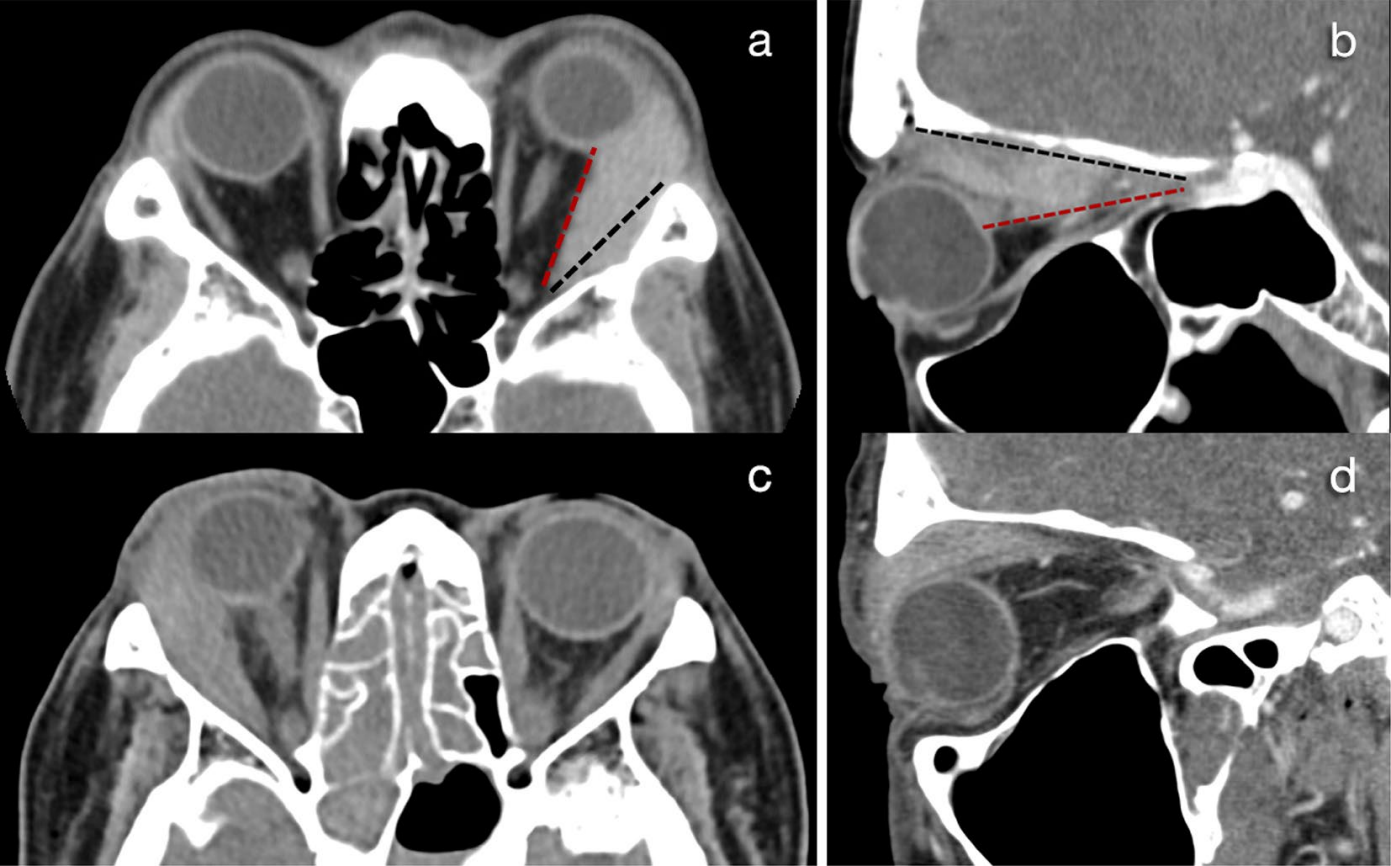
Representative images of the wedge signs. The presence of a triangle shape of tissue between the lateral rectus muscle (red line) and lateral orbital wall (black line) is termed a lateral wedge sign, the histologic diagnosis was mucosa-assisted lymphoid tissue (MALT) lymphoma (**a**). The triangle tissue between the superior rectus muscle (red line) and roof of the orbit (black line) is termed a superior wedge sign, the histologic diagnosis was adenoid cystic carcinoma (**b**). The lateral wedge sign in immunoglobulin G4-related disease (**c**). The superior wedge sign in reactive lymphoid hyperplasia (**d**).

Data collected included patient demographics, clinical presentations, orbital imaging findings, histopathologic diagnoses, and treatment modalities. We examined the presence of wedge signs in the lateral and superior spaces (Fig. 1) on orbital imaging to predict the possibility of adenoid cystic carcinoma (ACC) and lymphoma. The correlation between the radiologic features and each type of lacrimal gland tumor (ACC, lymphoma, and non-epithelial non-malignant tumors) was also investigated. Additionally, treatment outcomes, including prognosis and survival, were assessed. For selected patients who were initially diagnosed with nonspecific dacryoadenitis [idiopathic orbital inflammation (IOI)], the increment of data for the diagnosis of immunoglobulin G4-related disease (IgG4-RD) was added from a previously published study^12^. The primary outcome was to assess the clinical characteristics, radiologic features, and histopathologic findings of lacrimal gland tumors. The secondary outcome was to study the treatment outcomes and survival in patients with lacrimal gland malignancies. Treatment outcomes were evaluated based on clinical conditions and orbital imaging.

Data were analyzed using STATA version 14 (StataCorp LP, College Station, TX, USA). Descriptive statistics, including mean ± standard deviation, median, and frequency (%), were calculated. The chi-squared or Fisher’s exact test was used to analyze the relationships between categorical variables. The Mann–Whitney U or independent t-test was used to analyze the relationship between continuous variables. Associations between wedge signs and histologic diagnoses (ACC, lymphoma, and non-malignant non-epithelial tumor) were evaluated using multinomial logistic regression and expressed as relative risk ratios (RRRs). Overall survival (OS) was defined as the time between the date of diagnosis and the date of the last follow-up or death. Patients without a documented date of death were censored at the time of their last known follow-up. Survival curves were estimated using the Kaplan–Meier method and compared across groups using the log-rank test. Statistical significance was set at *p* < 0.05.

## Results

### Demographic data and clinicopathologic features

A total of 106 patients were histopathologically diagnosed during the study period; however, 27 patients were excluded because of a follow-up period of less than 3 months (22 patients) and lack of orbital imaging (5 patients). Finally, 79 patients (106 eyes) were included in the study, with a median follow-up of 51.5 months (range 3.0–233.0 months). The median age was 48.9 years (range 18.3–88.3 years), with 51.9% females. Most patients had symptoms lasting for more than 3 months (60.8%). Non-epithelial tumors of the lacrimal gland were reported in 77.2% of all cases. Patients with epithelial tumors of lacrimal glands were significantly more likely to have a longer mean duration of symptoms (26.3 months) and to be associated with diplopia (61.1%), whereas they were less likely to have lid swelling (61.1%), compared with those with non-epithelial tumors (7.3 months, 24.6%, and 96.7% respectively, *p* < 0.05 for each comparison).

The most common histologic diagnosis of all biopsied lacrimal glands was IgG4-RD [n = 23 (29.1%)], followed by lymphoma [n = 14 (17.7%)] and reactive lymphoid hyperplasia (RLH) [n = 14 (17.7%)]. Fifteen of the 61 patients with non-epithelial tumors of the lacrimal gland underwent repeat biopsies. Ten patients were reported to have different histology after the repeat biopsy: eight patients with initial findings of RLH (n = 4) and IOI (n = 4) had final results of IgG4-RD, and two patients initially diagnosed with RLH had lymphoma after re-biopsy. In five patients, the histopathologic diagnosis remained unchanged (3 RLH and 2 lymphoma).

Patients with ACC were significantly more likely to have a shorter duration of symptoms (median, 4 months) and proptosis (100.0%) than those with pleomorphic adenoma (median, 15 months and 33.3%; *p* = 0.040 and *p* = 0.009, respectively) (Table 1). Patients with lymphoproliferative disorders were more likely to be older (median, 63.4 years) and predominantly male (67.9%) than those with dacryoadenitis (median, 48.9 years and 39.4%; *p* = 0.044 and *p* = 0.027, respectively). Patients with IgG4-RD were older (median, 54.3 years) than those with nonspecific inflammation (36.2 years; *p* = 0.046). Patients with ACC (88.9%) were more likely to present with diplopia than those with lymphoma (0.0%; *p* < 0.001).

**Table 1:** Demographic and clinical characteristics of patients with lacrimal gland tumors.

Variables	ACC (n = 9)	Pleomorphic adenoma (n = 9)	*p* value	Lymphoma (n = 14)	RLH (n = 14)	*p* value	IgG4-RD (n = 23)	Nonspecific dacryoadenitis (n = 10)	*p* value
Age (years)									
Median (min, max)	46.90 (17.01, 69.41)	43.95 (24.35, 57.51)	0.310	63.27 (38.86, 82.68)	64.33 (2.79, 82.65)	1.000	54.31 (20.72, 88.29)	36.21 (18.29, 82.65)	0.046*
Sex, n (%)									
Male	5 (55.6)	1 (11.1)	0.131	11 (78.6)	8 (57.1)	0.420	9 (39.1)	4 (40.0)	1.000
Female	4 (44.4)	8 (88.9)		3 (21.4)	6 (42.9)		14 (60.9)	6 (60.0)	
Laterality, n (%)									
Unilateral	9 (100.0)	9 (100.0)	–	10 (71.4)	8 (57.1)	0.695	40 (43.5)	6 (60.0)	0.465
Bilateral	0 (0.0)	0 (0.0)		4 (28.6)	6 (42.9)		13 (56.5)	4 (40.0)	
Duration of symptom (months), median (min, max)	4 (1, 12)	15 (2, 240)	0.040*	3.5 (1, 24)	3 (1, 24)	0.501	5.0 (1, 36)	3 (1, 60)	0.228
Duration of symptoms (months)									
≤ 1	2 (22.2)	0 (0.0)	0.311	2 (14.3)	3 (21.4)	0.742	2 (8.7)	3 (30.0)	0.094
> 1–3	2 (22.2)	2 (22.2)		4 (28.6)	5 (35.7)		3 (13.0)	3 (30.0)	
> 3	5 (55.6)	7 (77.8)		8 (57.1)	6 (42.9)		18 (78.3)	4 (40.0)	
Clinical presentation									
Pain	2 (22.2)	3 (33.3)	1.000	3 (21.4)	3 (21.4)	1.000	9 (39.1)	3(30.0)	0.710
Lid swelling	6 (66.7)	5 (55.6)	1.000	12 (85.7)	14 (100.0)	0.481	23 (100.0)	10 (100.0)	-
Palpable mass	7 (77.8)	8 (88.9)	1.000	10 (71.4)	14 (100.0)	0.098	20 (87.0)	7 (70.0)	0.336
Proptosis	9 (100.0)	3 (33.3)	0.009*	11 (78.6)	6 (42.9)	0.120	15 (65.2)	4 (40.0)	0.257
Eyelid erythema	2 (22.2)	0 (0.0)	0.471	1 (7.1)	2 (14.3)	1.000	0 (0.0)	1 (10.0)	0.303
Diplopia	8 (88.9)	3 (33.3)	0.050	0 (0.0)	4 (28.6)	0.098	10 (43.5)	1 (10.0)	0.109
Decreased vision	4 (44.4)	1 (11.1)	0.294	1 (7.1)	2 (14.3)	1.000	2 (8.7)	1 (10.0)	1.000
Systemic involvement	2 (22.2)	0 (0.0)	0.471	3 (21.4)	1 (7.1)	0.596	2 (8.7)	1 (10.0)	1.000

*n* number, *ACC* adenoid cystic carcinoma, *RLH* reactive lymphoid hyperplasia, *IgG4-RD* immunoglobulin G4-related disease.

*Statistically significant.

### Radiologic features

The characteristics of orbital imaging in patients with pleomorphic adenoma were well-circumscribed, oval or round shape with a smooth surface, bone remodeling, and no adjacent tissue invasion; however, the ACC was ill-defined with a nodular surface, bony destruction, and adjacent tissue invasion (*p* < 0.05 for each comparison). We also examined the presence of the wedge sign in a radiologic study. The lateral wedge sign has been reported in ACC (66.7%), IgG4-RD (43.5%), lymphoma (35.7%), nonspecific dacryoadenitis (30.0%), and RLH (28.6%). The superior wedge sign was commonly found in ACC (55.5%), followed by lymphoma (21.4%), IgG4-RD (13.0%), RLH (7.1%), and nonspecific dacryoadenitis (0.0%). The superior wedge sign was significantly associated with ACC compared with non-malignant non-epithelial lacrimal gland tumors, with an RRR of 13.44 (Table 2).

**Table 2:** Wedge signs in lacrimal gland tumors.

Wedge sign	Non-malignant tumor (n = 47)	ACC (n = 9)	Lymphoma (n = 14)	*p* value	RRR for ACC^$^ (95% CI)	*p* value	RRR for lymphoma^$^ (95% CI)	*p* value
Lateral wedge, n (%)								
Absence	30 (63.8)	3 (33.3)	9 (64.3)	0.260	1	0.101	1	0.975
Presence	17 (36.2)	6 (66.7)	5 (35.7)		3.53 (0.78, 15.95)		0.98 (0.28, 3.40)	
Superior wedge, n (%)								
Absence	43 (91.5)	4 (44.4)	11 (78.6)	0.002*	1	0.002*	1	0.198
Presence	4 (8.5)	5 (55.6)	3 (21.4)		13.44 (2.54, 71.16)		2.93 (0.57, 15.07)	

*ACC* adenoid cystic carcinoma, *n* number, *RRR* relative risk ratio, *CI* confidence interval.

* Statistically significant.

^$^Compared with non-malignant tumors of the lacrimal gland, except pleomorphic adenoma.

### Treatment modalities and outcomes

Several treatment modalities exist for lacrimal gland tumors depending on the histopathologic diagnosis. In the dacryoadenitis group, including patients with IgG4-RD and nonspecific dacryoadenitis, corticosteroids were the main treatment modality (78.1%), with a starting dose of 0.6–1 mg/kg/day. The results showed that half of the patients with IgG4-RD had complete resolution (52.2%), and 47.8% had recurrence at a mean follow-up of 45.7 months. Sixty percent of the patients with nonspecific dacryoadenitis had complete resolution, with 30% showing recurrence and 10% showing no improvement at a mean follow-up of 56.0 months. Patients with pleomorphic adenoma who had undergone complete tumor excision had no recurrence at a mean follow-up of 26.5 months (range 4.0–114.5 months).

Fourteen patients who were diagnosed with RLH received systemic corticosteroids (n = 9), orbital radiation (n = 3), and observation (n = 2); 8 out of 14 cases had complete resolution, five had recurrence, and one had partial response during a mean follow-up of 42.5 months. Of the 14 patients with lymphoma, five received chemotherapy without rituximab during the study period. Of the three patients who received cyclophosphamide, vincristine, doxorubicin, and prednisolone regimen, one experienced complete resolution, one had a recurrence, and one did not improve. Of the two patients who received cyclophosphamide, vincristine, and prednisolone regimen, one experienced complete resolution and one had a recurrence. Five patients who underwent orbital radiation with a dose range from 3.0 to 3.6 grays, experienced complete resolution. Complete resolution was reported in 40% in the chemotherapy group and 100% in the radiation group (*p* value = 0.167). All patients received the treatment, seven experienced complete resolution, two had a recurrence at 17 and 41.5 months, and one did not improve during a mean follow-up of 56.8 months. Four patients with lymphoma who chose to observe, their clinical conditions did not improve.

**Figure 2:**
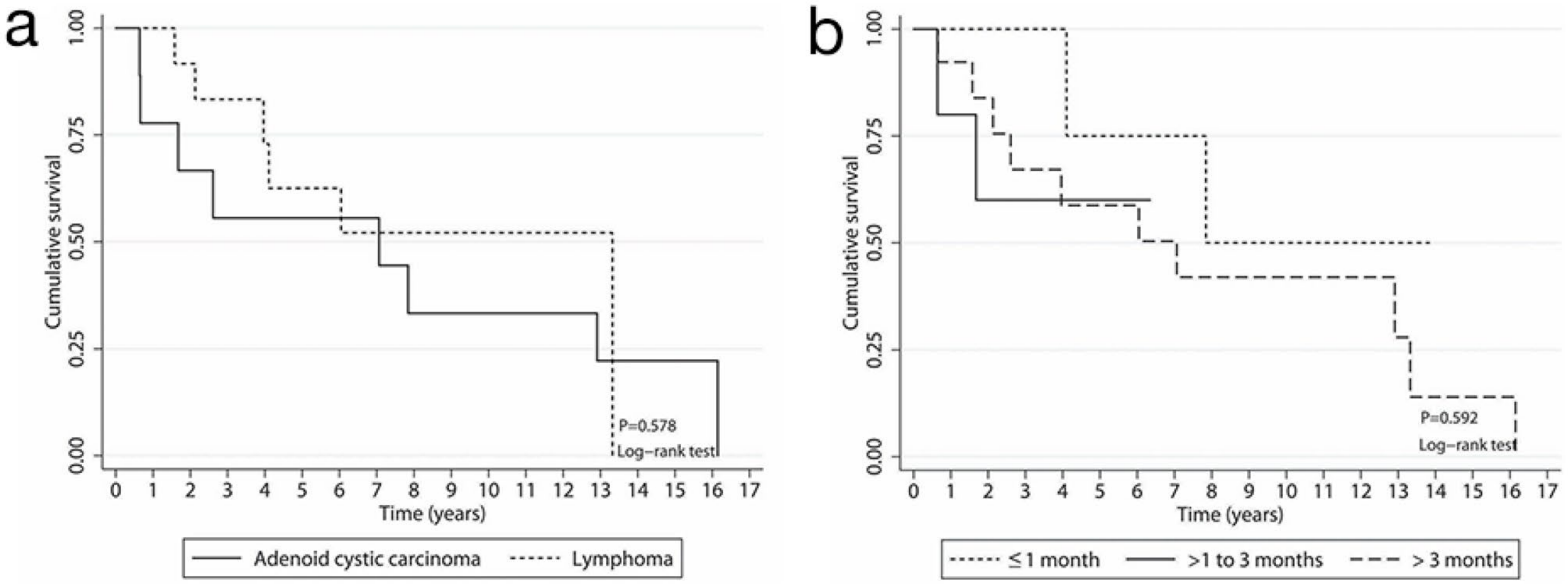
Kaplan–Meier curves. Overall survival according to histologic diagnosis (**a**) and symptom duration (**b**).

For patients with ACC, tumor (T) staging at initial diagnosis based on the eighth edition of the American Joint Committee on Cancer classification was assessed for epithelial lacrimal gland carcinoma as follows: T2c, three patients; T3c, one patient; and T4c, five patients. Initially, five of the nine patients underwent lateral orbitotomy with total tumor removal, three underwent orbital exenteration, and one missed the appointment for exenteration after an incisional biopsy. Finally, two of the five patients who had previously undergone lateral orbitotomy with total tumor removal underwent orbital exenteration. The mortality rates due to ACC and lymphoma were 0.126 and 0.081 per person-year, respectively. The median survival times of patients with ACC and lymphoma were 7.06 years and 13.32 years, respectively (Table 3). The Kaplan–Meier curves of lacrimal gland malignancy are shown in Fig. 2. The results showed no significant difference in OS between patients with ACC and those with lymphoma, in addition to variation in symptom duration.

**Table 3:** Overall survival of patients with malignant lacrimal gland tumors.

Characteristics	n/N	Rate/PY	Median	% of survival by year				*p* value	Crude HR	*p* value
				1	3	5	10			
Overall survival	14/23	0.101	7.06	90.9	71.5	60.0	43.0	–	–	–
Diagnosis										
ACC	8/9	0.126	7.06	77.8	55.6	55.6	33.3	0.578	1	0.579
Lymphoma	6/14	0.081	13.32	100.0	83.3	62.5	52.1		0.73 (0.24, 2.23)	
Sex										
Male	9/16	0.105	6.04	83.7	71.5	53.6	44.7	0.976	1	0.976
Female	5/7	0.097	7.84	100.0	71.4	71.4	35.7		1.02 (0.33, 3.13)	
Laterality										
Unilateral	11/19	0.093	7.84	89.5	72.3	65.7	44.4	0.405	1	0.410
Bilateral	3/4	0.158	3.96	100.0	66.7	33.3	33.3		1.74 (0.47, 6.51)	
Duration of symptoms (months)										
≤ 1	2/4	0.053	7.84	100.0	100.0	75.0	50.0	0.592	1	
> 1–3	2/6	0.146	–	80.0	60.0	60.0	60.0		2.65 (0.34, 20.53)	0.351
> 3	10/13	0.117	7.06	92.2	67.1	58.7	42.0		1.98 (0.43, 9.20)	0.384

*n* number of dead patients, *N* total number of patients, *PY* per year, *ACC* adenoid cystic carcinoma, *HR* hazard ratio.

*Statistically significant.

## Discussion

This retrospective study describes the clinicopathologic findings, radiologic features, and prognosis of biopsy-proven lacrimal gland tumors in southern Thailand. We found that approximately three-fourths were non-epithelial tumors (77.2%), and the other quarter was tumors of epithelial origin (22.8%). Patients with dacryoadenitis were younger and more likely to be female than those with lymphoproliferative disorders. The most common etiology in our study was dacryoadenitis (41.7%), which was similar to previous studies in Korea (52.6%)^10^, Australia (50%)^13^, and Singapore (46.4%)^9^. Since the lacrimal gland is often a target of the immune system, this causes autoimmune disease as well as the unique structure of the lacrimal gland. Thus, inflammation and lymphoproliferative disorder occur frequently in the lacrimal gland^14^.

Our study included different inclusion criteria from those of previous studies. Although most cases had a symptom duration of more than 3 months (60.8%), the proportions of histopathologic findings in the various ranges of symptom duration (≤ 1 month, > 1–3 months, > 3 months) was not significantly different. Most published reports included patients who had undergone lacrimal gland biopsy and had a clinical presentation for more than 3 months, defined as chronic lacrimal gland disease^9–11^. According to our results, even if the duration was ≤ 1 month, advanced-stage malignant tumors were still identified on histopathologic examination. Two of nine patients with ACC and 2 of the 14 patients with lymphoma had a symptom duration of ≤ 1 month. Since the duration of symptoms was subjective, the reason for the short duration in such malignant could be attributed to the factors like the patients were unaware or were not concerned about the abnormalities or the rapid progression of aggressive disease. Therefore, we suggest performing the orbital imaging urgently for all patients with lacrimal gland tumors for obtaining the provisional diagnosis. Our results encourage surgeons and patients to discuss early tissue biopsy before empirical treatment with systemic steroids because the clinical characteristics and orbital imaging findings cannot distinguish between nonspecific dacryoadenitis, IgG4-RD, RLH, lymphoma, and early ACC. Furthermore, most non-epithelial lacrimal gland tumors, including lymphoma, temporarily improve with systemic steroids^15,16^, which is the cause of delayed diagnosis and possibly affects the histologic diagnosis as well^17^.

We found that patients with non-epithelial tumors had a shorter duration of symptoms (mean, 7.3 months) compared with those with epithelial tumors (26.3 months; *p* = 0.019), more commonly presented with lid swelling, and were less likely to have diplopia. Owing to the proportion of epithelial tumors of the lacrimal gland, approximately 50% were pleomorphic adenomas, which are slowly progressive masses with a median symptom period of 15 months (longest duration, 10 years). However, patients with ACC had a symptom duration of 4 months.

Our study compared the characteristics of patients with lymphoproliferative disorders and those with dacryoadenitis. The median age in the dacryoadenitis group was significantly younger, and the female sex predominated when compared with those in the lymphoproliferative disorders group, which was similar to the results of previous studies^9,10,18^. The most common sites of IgG4-RD and nonspecific dacryoadenitis are the lacrimal gland^14,19^. Our study revealed that patients with IgG4-RD were older than those with nonspecific dacryoadenitis. However, different features between IgG4-RD and nonspecific dacryoadenitis in the orbit have been reported regarding frequent bilateral disease and submandibular nodal involvement in IgG4-RD^12,20^. Our study did not reveal IgG4-RD-associated systemic involvement; this might be an underestimation of the systemic investigation, as especially over the past decade, IgG4-RD is not a well-understood disease. Recently, IgG4-RD has been more frequently identified as the specific cause of lacrimal gland inflammation^21^. The diagnosis of IgG4-related ophthalmic disease is not necessary to evaluate the systemic association. However, IgG4-RD of the lacrimal gland was associated with systemic involvement in 52.4% of patients^10^. Since the treatment of choice in IgG4-RD is systemic corticosteroids, early non-symptomatic organ involvement would be treated regardless. We also compared the clinical features of RLH and lymphoma, both of which presented in the sixth decade without any significantly different clinical characteristics. Tissue biopsy plays an important role in establishing a definitive diagnosis of RLH and lymphoma.

Tissue diagnosis of lacrimal gland tumor is simple and safe, and is performed mostly under local anesthesia. However, surgeons must avoid performing lacrimal gland biopsy via a transconjunctival approach, which can result in lacrimal duct orifice injury. In addition, it is beneficial to preserve the lacrimal artery, which can be utilized for intra-arterial chemotherapy in patients with suspected ACC^22^. In the case of a young woman who presented with a painful lacrimal gland enlargement within 2–3 days of the onset of symptoms, the diagnosis was likely to be typical dacryoadenitis; performing tissue biopsy was not necessary, and systemic steroid treatment was the treatment of choice.

The presence of the wedge sign in the radiologic study was consistent with the findings of a previous study by Lorenzano and Rose^23^ in which it was suggested that the sign is mostly found in lacrimal gland carcinoma. Our study showed that ACC was strongly associated with the wedge sign, and the presence of a superior wedge could significantly predict ACC. All patients with lacrimal gland tumors should undergo computed tomography or magnetic resonance imaging to properly evaluate the nature of the disease because ACC has a poor prognosis and a high risk of brain invasion. However, our study demonstrated that neither the lateral nor superior wedge signs could indicate lymphoma compared with non-malignant lacrimal gland tumors. As an infiltrative lesion along the globe and bone, the wedge sign can also be found in patients with severe orbital inflammation. Wedge signs in imaging can differ between ACC and non-epithelial tumors of the lacrimal gland, including lymphoma. This information can help the surgeon to make a decision for urgent tissue biopsy in patients with suspected ACC where there is limited medical resource.

In southern Thailand, our center is the major tertiary care center, established as an oculoplastic center in 2004; hence, patients with lacrimal gland tumors from 14 provinces have been referred to our center. Approximately 2000 patients visit our oculoplastic clinic, and 340 undergo operations annually. In 2016, the oculoplastic center was set up in Surat Thani Province, and in 2019, it was set up in Phuket Province. We suggest that the primary care doctor should send the orbital imaging urgently. Patients who need to be referred for tissue diagnosis included atypical clinical presentation, long duration of symptoms > 3 months, recurrence, non-response to treatment, or positive superior wedge sign.

In our study, 50–60% of patients with dacryoadenitis had complete resolution, with a high rate of recurrence on tapering or cessation of steroids. Although IgG4-RD of the lacrimal gland increased, the main treatment was systemic steroids. Patients with pleomorphic adenoma had good disease prognosis after complete tumor excision, including its capsule, through a lateral orbitotomy. The result was satisfactory, showing complete resolution without recurrence in all patients, which was in line with studies from Spain^24^ and the Netherlands^25^.

In our study, all patients did not receive rituximab or other targeted therapy; thus, the treatment outcome of chemotherapy had a lower success rate when compared with findings of the study from Korea^26^. The prognosis observed from the percentage of OS between the two groups of patients with ACC and lymphoma was not significantly different (*p* = 0.578). In our study, the 5-year OS rates of patients with ACC and lymphoma were 55.6% and 62.5%, respectively. The 10-year OS rates of patients with ACC and lymphoma were 33.3% and 52.1%, respectively, which were similar to a United States study comprising Caucasian, African American, Asian, and Pacific Islander populations that demonstrated 10-year OS rates for patients with ACC and lymphoma of 38.7% and 55.1%, respectively^27^. Both ACC and lymphoma of the lacrimal gland have a poor prognosis.

This study had some limitations. The retrospective design resulted in incomplete data due to the absence of a data record form. We enrolled patients who underwent tissue biopsy for lacrimal gland mass from 2004 when the oculoplastic unit was established. Thus, newer treatment modalities were used at the early stages of the study. Data were also collected from a single tertiary center in southern Thailand, which may not be representative of the Thai population. A multicenter study may be suggested to provide further diagnostic guidance for appropriate management, prognosis, and survival rate of each lacrimal gland malignancy gained from this study, which would be useful for future research.

In summary, the most common histologic diagnosis was dacryoadenitis with IgG4-RD predominance. Patients with ACC had a significantly shorter symptom duration and were more likely to have proptosis than patients with pleomorphic adenoma. IgG4-RD was mostly observed in older patients. Patients with ACC were more likely to present with diplopia than with lymphoma. Radiologic features, including shape, surface, bony changes, and wedge sign, can guide provisional diagnosis and proper management. Even though patients present with a short symptom duration, atypical clinical characteristics, or positive superior wedge sign, a histologic diagnosis from lacrimal gland biopsy, which is crucial for appropriate treatment, is not a complicated procedure and has low morbidity.

## Data Availability

All data are available upon reasonable request to the corresponding author.
